# Applicability of propidium monoazide (PMA) for discrimination between living and dead phytoplankton cells

**DOI:** 10.1371/journal.pone.0218924

**Published:** 2019-06-25

**Authors:** Sungbae Joo, Phillip Park, Sangkyu Park

**Affiliations:** 1 Division of Basic Research, National Institute of Ecology, Seocheon, Republic of Korea; 2 Department of Biological Science, Ajou University, Suwon, Republic of Korea; INRA, FRANCE

## Abstract

Propidium monoazide (PMA) is a highly selective dye that penetrates only membrane-compromised, dead microbial cells and inhibits both DNA extraction and amplification. PMA has been widely used for discrimination between living and dead microbial cells; however, the application of PMA in phytoplankton studies has been limited. In this study, we attempted to evaluate its applicability for the discrimination of viable phytoplankton. We tested PMA on seven phytoplankton species, *Microcystis aeruginosa*, *Anabaena* sp., *Aphanizomenon* sp., *Synechocystis* sp., *Cryptomonas ovata*, *Scenedesmus obliquus*, and *Nitzschia apiculata* as representatives of the major phytoplankton taxa Cyanobacteria (first four species), Chlorophyta, Cryptophyta, and Bacillariophyta, respectively. Our results showed that application of PMA to phytoplankton living in freshwater has the potential to distinguish viable from dead cells as in microbial studies. Particularly, PMA differentiated viable from dead cells in cyanobacterial species rather than in other phytoplankton taxa under our experimental conditions. However, our results also showed that it may be necessary to adjust various conditions affecting PMA treatment efficiency to expand its applicability to other phytoplankton. Although all factors contributing to the effects of PMA could not be evaluated, our study showed the applicability of PMA-based molecular approaches, which can be convenient quantitative methods for distinguishing living from dead phytoplankton in freshwater ecosystems. Setting optimal treatment conditions for other phytoplankton species may increase the efficacy of PMA-based molecular approaches.

## Introduction

Discrimination between viable and dead phytoplankton cells can provide novel perspectives in ecological research [1–3]. In terms of energy flows, viability of phytoplankton could serve as one of the main factors determining seston food quality for zooplankton [4–6]. For example, some calanoids, such as *Eucalanus pileatus*, can discriminate between the food quality of different particles and prefer living rather than dead phytoplankton cells [7, 8]. In addition, detection of viable phytoplankton species is essential to prevent the spread of non-indigenous species transported in the ballast water of ships [9, 10]. Various methods have been tested to reduce the number of viable organisms in ballast water, and the potential survival and regrowth of harmful organisms should be measured after treatment in accordance with the guidelines of the Ballast Water Convention adopted by the International Maritime Organization (IMO) [11–13].

Regarding methodological aspects, many attempts to discriminate between living and dead phytoplankton have been undertaken. The general method used to determine cell viability is plating the culture and subsequent cells for counting [14, 15]. However, this method is time-consuming because it requires at least 1 week for plate preparation and cell identification. As an alternative, fluorescence staining, including microscopic and flow cytometric approaches, has been widely used for cell viability determination [16, 17]. These approaches have the advantages of being less time-consuming and more reliable than the previous methods, but have lower identification resolution than DNA-based identification. A cell digesting assay (CDA), a non-staining method, was used to identify living cells in cultured and natural phytoplankton populations [18, 19]. CDA is based on the selective digestion of dead cells by digestive enzymes (DNAse I and trypsin) while leaving living cells intact and countable [15]. However, this method also requires additional microscopic observation or flow cytometry for counting and identifying living cells.

Propidium monoazide (PMA) is a high-affinity photoreactive DNA-intercalating agent [20]. PMA dye is highly membrane impermeable in living cells and only permeable in membrane-compromised dead cells. It selectively combines only with exposed DNA from dead cells. PMA preferentially binds to double-stranded DNA with high affinity upon photolysis and prevents DNA extraction or amplification by PCR [20]. Recently, many studies using PMA for discrimination between living and dead cells have been reported in various fields [21–24]. Roth et al. [25] proposed the applicability of PMA for discerning viable benthic diatom community structure. Nocker et al. [26] showed that PMA treatment in seawater samples reduced the relative abundance of chloroplasts in heat-exposed samples using 454 pyrosequencing. However, there was no satisfactory explanation regarding phytoplankton. The authors documented the largest and most obvious effect involved in sequences corresponding to cyanobacterial rRNA or plastids in eukaryotic algae because of the sequence similarity of the target genes.

Our study aimed to evaluate the potential applicability of PMA to various phytoplankton species. We tested the discriminatory ability of PMA in seven different phytoplankton species as representatives of four phyla (four cyanobacterial species and one species each for Chlorophyta, Cryptophyta, and Bacillariophyta). In addition, we examined whether PMA treatment could reflect defined proportions of living and dead cells of *Microcystis aeruginosa*, a major cyanobacterial species causing water blooms, as the representative strain.

## Materials and methods

### Sample preparation and culture conditions

Two phytoplankton species, *M*. *aeruginosa* (UTEX 2385) and *Cryptomonas ovata* (UTEX LB2783), were obtained from the Culture Collection of Algae at the University of Texas at Austin (UTEX, USA). In addition, *Scenedesmus obliquus* (KMMCC-1234) and *Nitzschia apiculata* (KMMCC-1209) were obtained from the Korea Marine Microalgae Culture Center (KMMCC, South Korea). Three cyanobacterial species, *Anabaena* sp., *Aphanizomenon* sp., and *Synechocystis* sp. were isolated from the Han River, South Korea by the Eco-healing Laboratory of Konkuk University. All phytoplankton species was cultured in Bold 3N medium and maintained in the exponential growth phase by weekly sub-culturing at 25°C under fluorescent lights at 40 μmol photons m^-2^ s^-1^ in a light/dark cycle of 16:8 h. They were maintained at 25°C in a temperature-controlled chamber under a 16:8 h light:dark cycle.

### Killing conditions

To set various ratios of experimental conditions, we killed the cultured phytoplankton before PMA treatment. Six phytoplankton species, except for *N*. *apiculata*, were exposed to 60°C at 750 rpm for 5 min using the Thermomixer Comfort (Eppendorf, Germany). As an alternative to the heat exposure treatment, *N*. *apiculata* was killed by exposure to liquid nitrogen for 5 min because heat exposure, as observed in a preliminary experiment, causes loss of DNA.

### PMA treatment

To concentrate the phytoplankton species, 400 ml of cultured phytoplankton strains was transferred into eight 50 ml conical tubes and harvested by centrifugation at 3,000 × *g* for 15 min. After removing the supernatant, the cells were resuspended with 500 μl of distilled water and transferred to 2 ml Eppendorf tubes. PMA was mixed with 500 μl of each sample at a final concentration of 50 μM. After incubation for 5 min in the dark at room temperature, the samples were placed horizontally on ice and then light-exposed for 5 min using 300W × 2 halogen lamps (Osram 64705) at a distance of approximately 20 cm from the sample tubes. Each sample was harvested by centrifugation (5,000 × *g* for 5 min) before DNA extraction.

### DNA isolation, PCR amplification, and quantification

Genomic DNA was extracted using the FastDNA SPIN Kit for Soil (MP Biomedicals, USA) according to the manufacturer’s protocols, except for the lysis step. For sufficient homogenization, we increased the shaking on a Mixer Mill (Retsch, Germany) to 20 Hz for 2 min after 29 Hz for 1 min during the homogenization step. Except for cyanobacterial species, both 18S ribosomal RNA (18S rRNA) from eukaryotic algae and 16S ribosomal RNA (16S rRNA) of plastids were used as the target genes to compare the effect of PMA based on target regions. In each PCR amplification, 1 μl of extracted DNA was added to 24 μl of the amplification mixture, resulting in final concentrations of 1× Ex *Taq* Buffer, 1.5 mM MgCl_2_, 0.2 mM dNTPs, 0.2 μM of each primers (18S rRNA: primer A, 5′-AAC CTG GTT GAT CTT GCC AGT-3′ and primer B, 5′-TGA TCC TTC TGC AGG TTC ACC TAC-3′, [27]; 16S rRNA: PSf, 5′-GGG ATT AGA TAC CCC WGT AGT CCT-3′ and Ur, 5′-TAC GGY TAC CTT GTT ACG ACT T -3′, [28], and 1 U of Ex *Taq* DNA polymerase (Takara, Japan), in a final reaction volume of 25 μl. PCR conditions were as follows: an initial denaturation at 95°C for 4 min, 25 cycles of denaturation at 95°C for 30 s; annealing at 60°C for 30 s; elongation at 72°C for 1 min 30 s, and a final extension step at 72°C for 7 min. In the case of *Anabaena* sp., the amplification cycles were increased to 30 because of the low initial DNA concentration. DNA quantification was conducted using the PicoGreen quantification solution (Molecular Probes Inc., USA) and a SPECTRA max GEMINI XS microplate spectrofluorometer (Molecular Devices, Sunnyvale, CA, USA).

### Quantitative PCR

Quantitative PCR (qPCR) was conducted in mixtures with defined ratios of living and dead *M*. *aeruginosa* targeting the 16S rRNA gene. Two microliters of extracted DNA was added to 18 μl of the PCR amplification mixture containing iQ SYBR Green Supermix (Bio-Rad, USA) and 0.2 μM of each primers in a final reaction volume of 25 μl. PCR conditions were the same as described above, except that the amplification cycles were extended to 45 cycles. PCR was performed using a CFX96 C1000 thermal cycler (Bio-Rad, USA).

### Statistical analysis

The correlation between the DNA concentration and the cycle of threshold (*C*_t_) value after qPCR was calculated using the R software [29]. All statistical analyses were performed using the R software (stats-package) [29].

## Results

### Effects of PMA treatment on various phytoplankton species

DNA yield was measured for seven different phytoplankton species as representatives of phytoplankton phyla after PMA treatment (Table 1). Regardless of the target genes, PMA treatment significantly reduced the DNA yields in dead phytoplankton (Table 1). DNA yields in PMA-treated dead cells were less than 10% in most cases, except for two species, *N*. *apiculata* (11.1% and 26.5% yield in both target regions, respectively) and *Synechocystis* sp. (19.8% yield in the case of 16S rRNA). In most cyanobacterial species, DNA yield was slightly reduced less than 3% for PMA-treated living cells (Table 1). However, one cyanobacterial species, *Synechocystis* sp., showed a 14.2% decrease as compared with non-PMA treatment conditions (Table 1). In case of *C*. *ovata* and *N*. *apiculate*, DNA yields under PMA-treated living cell conditions decreased more than 25% regardless of the target regions (Table 1).

**Table 1:** Effects of PMA treatment on the relative DNA yield of living and dead cells from five phytoplankton species after PCR.

Species	16S rRNA genes				18S rRNA genes			
	L	L+P	D	D+P	L	L+P	D	D+P
*Anabaena* sp.	100(1.8)	99.2(2.9)	99.9(2.2)	3.8(0.2)				
*Aphanizomenon* sp.	100(9.4) ^a^	97.8(9.2) ^a^	112.9(6.9) ^a^	7.2(0.19) ^b^				
*M*. *aeruginosa*	100(2.9) ^a^	97.3(3.3) ^a^	95.0(1.0) ^ab^	3.3(0.1) ^b^				
*Synechocystis* sp.	100(6.6) ^a^	85.8(4.7) ^a^	94.2(3.5) ^a^	19.8(1.8) ^b^				
*C*. *ovata*	100(8.4) ^a^	70.0(2.2) ^ab^	92.4(9.6) ^a^	5.1(0.4) ^b^	100(2.5) ^a^	73.1(13.1) ^ab^	96.2(2.6) ^a^	2.9(0.2) ^b^
*S*. *obliquus*	100(17.0) ^a^	114.3(5.3) ^a^	86.5(19.0) ^ab^	5.9(2.3) ^b^	100(2.6)	99.8(9.2)	94.2(14.4)	3.0(0.0)
*N*. *apiculata*	100(8.5) ^a^	54.9(12.6) ^ab^	79.4(0.7) ^a^	11.1(1.1) ^b^	100(17.5) ^a^	60.9(8.5) ^ab^	84.8(11.0) ^a^	26.5(1.8) ^b^

The effect of PMA was tested using two different target genes: 18S rRNA from eukaryotic algae and cyanobacterial, and plastidial 16S rRNA. In the case of the four cyanobacterial species, PMA efficacy was tested only on cyanobacterial 16S rRNA. Parentheses represent the standard deviations from three independent replicates. L: only living organisms; D: dead organisms; P: PMA dye treatment.

The significant differences between treatment groups were analyzed by Kruskal–Wallis test with post hoc Dunn’s multiple comparisons tests and expressed using different letters (*P* < 0.05).

**Fig 1:**
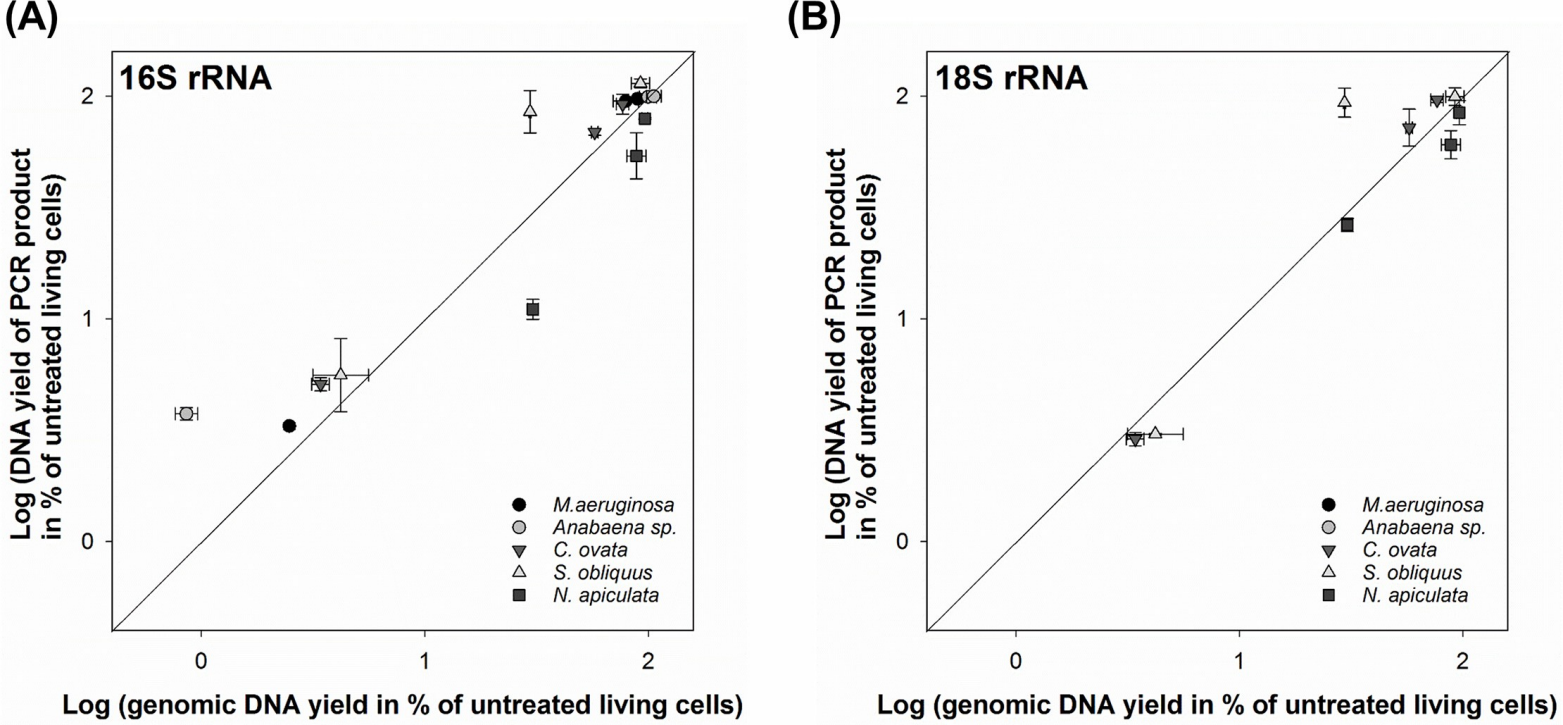
Relationship between the percentage of the amplified DNA yield and genomic DNA yield after PMA treatment. The line represents the identical ratio, and error bars represent the standard deviations from three independent replicates. (A) Amplified cyanobacterial and plastidial 16S rRNA and (B) amplified 18S rRNA from eukaryotic algae. The results of two cyanobacterial species, *Aphanizomenon* sp. and *Synechocystis* sp., are not represented because genomic DNA concentrations of both species were not measured during the experiments.

DNA yield of PCR products were generally well reflected in those of genomic DNA measured after the DNA extraction process and PMA treatment (Fig 1). However, some species also exhibited skewed ratios based on genomic DNA yield or the target gene. In case of *Anabaena* sp., DNA yield of PCR products were slightly overestimated under low gDNA yield conditions; however, the same for *S*. *obliquus* was overestimated under high gDNA yield conditions in both target genes.

### Effects of PMA treatment on the defined ratios of living and dead *M*. *aeruginosa*

**Fig 2:**
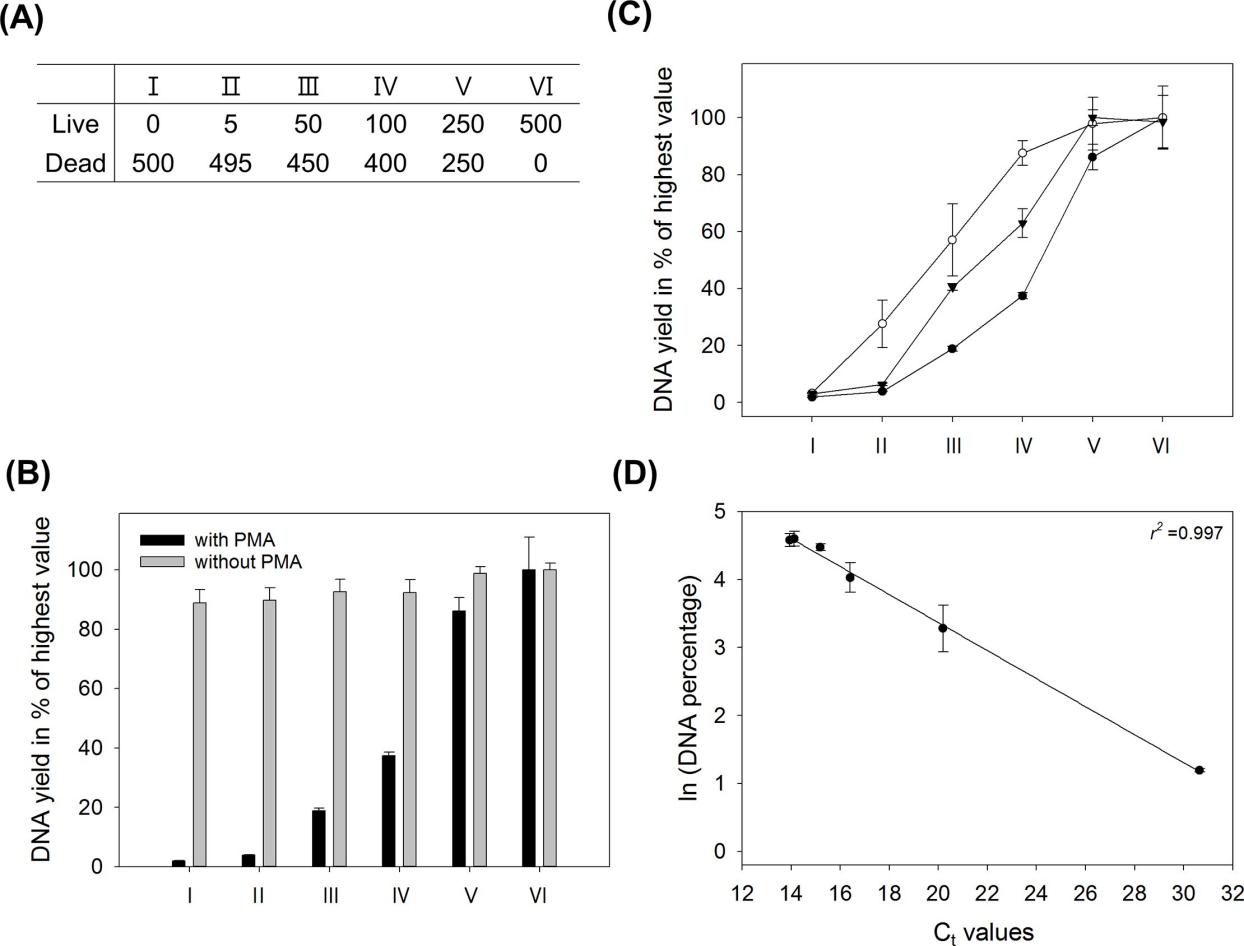
Effect of PMA treatment on the genomic DNA yield and PCR quantification of defined ratios of viable and heat-killed *M*. *aeruginosa*. The error bars represent the standard deviations from three independent replicates. (A) Mixing ratios of viable and heat-killed *M*. *aeruginosa* are as follows: living cells represent 0%, 1%, 10%, 20%, 50%, and 100% of the total cells (B) Genomic DNA yield (in percent) of the highest value according to the mixing ratio. Black bar: PMA treatment; Grey bar: non-PMA treatment. (C) PCR amplification bias according to template DNA concentration. Closed circle: Genomic DNA yield (in percent) of the highest value according to the mixing ratio in (B); Open circle: DNA yield obtained in a PCR with undiluted template DNA; Inverted closed triangle: DNA yield obtained in a PCR with diluted template DNA(1/10 dilution). (D) Correlation between normalized DNA concentrations and the corresponding *C*_t_ values after qPCR: y = −0.206x + 7.488, *r*^2^ = 0.997, *P* < 0.001.

Total DNA yield was measured in mixtures with defined ratios of living and dead *M*. *aeruginosa* after PMA treatment (Fig 2A). The genomic DNA yield increased with increasing proportions of living *M*. *aeruginosa* upon PMA treatment (Fig 2B). However, in most conditions, DNA yield tended to be overestimated compared to the defined ratios (Fig 2B). The DNA yield after PCR showed similar increasing patterns at high genomic DNA concentrations after PMA treatment, whereas they were overestimated when the percentage of living cells was low (Fig 2C). However, PCR bias was reduced by decreasing the initial DNA concentration (1/10 dilution) in the PCR (Fig 2C). Quantitative PCR showed that the *C*_t_ values after PMA treatment decreased with increases in the proportions of living cells. The DNA yield and *C*_t_ values showed a significantly linear correlation: *y* = −0.206*x* + 7.488, *r*^2^ = 0.997, *P* < 0.001 (Fig 2D).

## Discussion

Until recently, PMA treatment has been used in microbiological studies, especially for detecting viable bacteria, but a few studies have applied PMA dye for phytoplankton in freshwater ecosystems. The main purpose of this study was to evaluate the potential applicability of PMA for discriminating living phytoplankton from four major phytoplankton phyla in freshwater ecosystems. Our results showed that application of PMA for phytoplankton in freshwater can potentially distinguish viable from dead cells as in microbial studies. In particular, PMA treatment could differentiate viable from dead cells in cyanobacterial species rather than other phytoplankton taxa under our experimental conditions. However, to apply PMA to all phytoplankton species, it may be necessary to adjust various conditions affecting PMA treatment efficiency.

Regarding methodological aspects, the best advantage of PMA application for phytoplankton studies is that it can be directly applied downstream of molecular approaches, such as qPCR, denaturing gradient gel electrophoresis (DGGE), microarray analysis, and high throughput sequencing [26, 30–33]. A recent study compared commonly used techniques to identify living and/or dead cells and those applicable to microbial communities [34]. They explained that PMA application is compatible with many DNA-based techniques and relatively easy and fast to perform compared with other viability dyes. However, other morphological character-based methods, which are commonly used in phytoplankton studies, such as cell staining methods and CDA, are mainly focused on cell counting and quantification of living cells and have limitations regarding the discrimination of viable cells. These strategies might show relatively lower identification resolution than molecular-based approaches because of ambiguities in the fluorescence signals for living and dead cells [19, 35]. In particular, auto fluorescence of chlorophyll could disrupt the recognition of the fluorescent signals of the target cells (living or dead cells) by dyes and cause quantitative errors in flow cytometry approaches.

Fittipaldi et al. [36] reviewed the current knowledge on the use of viability dyes containing PMA and reported that the length of the target gene can influence the inhibition effects of viability dyes. Previous studies on bacteria reported that increasing PCR amplicon length efficiently caused the exclusion of dead cell signals because long PCR amplicon length increases the probability of PMA binding to the target DNA in dead cells [37–40]. Although we did not compare the effect of PMA based on length differences, primer sets used in this study amplified long DNA fragments (approximately 1.6 kb from 18S rRNA and longer than 700 bp from 16S rRNA) compared to those employed in previous studies. We considered that it was sufficient to overcome the limitation of short length effects.

Our results suggest that applying PMA is sufficient to screen for viable cells, especially in cyanobacterial species. However, our results also showed limitations wherein PMA treatment reduced DNA yields from non-dead cells up to 54.9% of untreated living cells, especially in diatom species. We assumed that these observations are due to the limitations of different dynamics of cell death depending on phytoplankton species in the experimental design. Lee and Rhee [35] mentioned that the dynamics of cell death may vary between phytoplankton species. Likewise, phytoplankton cell mortality can be affected by the growth stage and culture conditions [41]. Even though we cultured all the phytoplankton cells under appropriate culture conditions and used only cells in the exponential phase, the actual growth rates and cell mortalities in each phase might vary among the phytoplankton species during the experiment. Likewise, the membrane integrity and susceptibility to stress might vary among species and could affect the uptake of PMA because PMA treatment can only exclude membrane-compromised cells [17]. Additionally, Nocker and Camper et al. [42] noted differences in the killing efficacy by various killing methods, which induce membrane damage in their mini review. This means that heat-killing methods used in this study may induce different killing efficacy depending on the susceptibility of different phytoplankton species. For diatom species, we used exposure to liquid nitrogen as the killing method, whereas we used heat-killing methods for the other phytoplankton taxa. In preliminary experiments, heat-killing methods under our experimental condition showed low killing efficacy than exposure to liquid nitrogen for diatoms. In a recent study, diatom cells were successfully killed with heat-killing methods, but the temperature was increased to 80°C and exposure time to 2 h [25]. To apply PMA assays for other phytoplankton species, it may be necessary to validate treatment conditions, which influence the efficacy of PMA containing physical and chemical factors, such as incubation time and temperature, light exposure time, turbidity, and pH [43].

In general, the 18S rRNA gene has been widely used to study eukaryotic microorganisms, and considerable sequence information has been accumulated in public sequence databases [44–46]. The “universal” 18S rDNA primers used in this study can target all the phototrophic eukaryotes but cannot detect cyanobacterial species [28]. However, the phyto-specific (PS) primers used in this study were designed to recover the diversity of cyanobacterial species without detecting most of the non-photosynthetic bacteria. They also selectively amplify a broad diversity of microalgae in mixed environmental samples, while reducing the number of bacterial species detected [28, 47]. For this reason, at least with respect to identifying cyanobacterial species, the PS primers may have been more appropriate for the selective detection of cyanobacterial species in environmental samples.

To date, despite the many achievements of PMA-based methods in microbial studies, PMA has rarely been used for phytoplankton studies in freshwater ecosystems. Our results showed that PMA assays can be a novel approach that is most effective in discriminating living and dead cyanobacterial species. Although all the factors contributing to the effect of PMA could not be evaluated in this study, further studies overcoming the current limitations and setting optimal treatment conditions for other phytoplankton species will increase the applicability of PMA-based molecular approaches as convenient and quantitative methods for understanding the dynamics of phytoplankton community structure.

## References

[1] Jassby AD, Goldman CR. Loss rates from a lake phytoplankton community1. Limnology and Oceanography. 1974;19(4):618–27.

[2] Knoechel R, Kalff J. An in situ study of the productivity and population dynamics of five freshwater planktonic diatom species. Limnology and Oceanography. 1978;23(2):195–218.

[3] Reynolds C, Thompson J, Ferguson A, Wiseman S. Loss processes in the population dynamics of phytoplankton maintained in closed systems. Journal of Plankton Research. 1982;4(3):561–600.

[4] Brett MT, Müller‐Navarra DC, Sang‐Kyu P. Empirical analysis of the effect of phosphorus limitation on algal food quality for freshwater zooplankton. Limnology and Oceanography. 2000;45(7):1564–75.

[5] Müller-Navarra D, Lampert W. Seasonal patterns of food limitation in Daphnia galeata: separating food quantity and food quality effects. Journal of Plankton Research. 1996;18(7):1137–57.

[6] Sterner RW, Schulz KL. Zooplankton nutrition: recent progress and a reality check. Aquatic Ecology. 1998;32(4):261–79.

[7] Cowles T, Olson R, Chisholm S. Food selection by copepods: discrimination on the basis of food quality. Marine Biology. 1988;100(1):41–9.

[8] Donaghay P, Small L. Food selection capabilities of the estuarine copepod Acartia clausi. Marine Biology. 1979;52(2):137–46.

[9] Cariton JT, Geller JB. Ecological roulette: the global transport of nonindigenous marine organisms. Science. 1993;261(5117):78–82. 10.1126/science.261.5117.78 17750551

[10] Williams R, Griffiths F, Van der Wal E, Kelly J. Cargo vessel ballast water as a vector for the transport of non-indigenous marine species. Estuarine, Coastal and Shelf Science. 1988;26(4):409–20.

[11] Liebich V, Stehouwer PP, Veldhuis M. Re-growth of potential invasive phytoplankton following UV-based ballast water treatment. Aquatic Invasions. 2012(1).

[12] Reavie ED, Cangelosi AA, Allinger LE. Assessing ballast water treatments: Evaluation of viability methods for ambient freshwater microplankton assemblages. Journal of Great Lakes Research. 2010;36(3):540–7.

[13] Tsolaki E, Diamadopoulos E. Technologies for ballast water treatment: a review. Journal of Chemical Technology & Biotechnology. 2010;85(1):19–32.

[14] Schulze K, López DA, Tillich UM, Frohme M. A simple viability analysis for unicellular cyanobacteria using a new autofluorescence assay, automated microscopy, and ImageJ. BMC biotechnology. 2011;11(1):118. 22129198 10.1186/1472-6750-11-118 PMC3247844

[15] Zetsche E-M, Meysman FJ. Dead or alive? Viability assessment of micro-and mesoplankton. Journal of Plankton Research. 2012;34(6):493–509.

[16] Marie D, Simon N, Vaulot D. Phytoplankton cell counting by flow cytometry. Algal culturing techniques. 2005;1:253–67.

[17] Veldhuis MJ, Kraay GW, Timmermans KR. Cell death in phytoplankton: correlation between changes in membrane permeability, photosynthetic activity, pigmentation and growth. European Journal of Phycology. 2001;36(2):167–77.

[18] Agustí S, Sánchez MC. Cell viability in natural phytoplankton communities quantified by a membrane permeability probe. Limnology and Oceanography. 2002;47(3):818–28.

[19] Darzynkiewicz Z, Li X, Gong J. Assays of cell viability: discrimination of cells dying by apoptosis. Methods in cell biology. 41: Elsevier; 1994 p. 15–38. 7861963 10.1016/s0091-679x(08)61707-0

[20] Nocker A, Cheung C-Y, Camper AK. Comparison of propidium monoazide with ethidium monoazide for differentiation of live vs. dead bacteria by selective removal of DNA from dead cells. Journal of microbiological methods. 2006;67(2):310–20. 10.1016/j.mimet.2006.04.015 16753236

[21] Miotto P, Bigoni S, Migliori GB, Matteelli A, Cirillo DM. Early tuberculosis treatment monitoring by Xpert MTB/RIF. European Respiratory Journal. 2012;39(5):1269–71. 10.1183/09031936.00124711 22547737

[22] Rogers GB, Stressmann FA, Koller G, Daniels T, Carroll MP, Bruce KD. Assessing the diagnostic importance of nonviable bacterial cells in respiratory infections. Diagnostic microbiology and infectious disease. 2008;62(2):133–41. 10.1016/j.diagmicrobio.2008.06.011 18692341

[23] Soejima T, Minami J, Iwatsuki K. Rapid propidium monoazide PCR assay for the exclusive detection of viable Enterobacteriaceae cells in pasteurized milk. Journal of dairy science. 2012;95(7):3634–42. 10.3168/jds.2012-5360 22720921

[24] Wang L, Li Y, Mustapha A. Detection of viable Escherichia coli O157: H7 by ethidium monoazide real‐time PCR. Journal of applied microbiology. 2009;107(5):1719–28. 10.1111/j.1365-2672.2009.04358.x 19457030

[25] Roth P, Hill-Spanik KM, McCurry C, Plante C. Propidium monoazide-denaturing gradient gel electrophoresis (PMA-DGGE) assay for the characterization of viable diatoms in marine sediments. Diatom Research. 2017;32(3):341–50.

[26] Nocker A, Richter-Heitmann T, Montijn R, Schuren F, Kort R. Discrimination between live and dead cells in bacterial communities from environmental water samples analyzed by 454 pyrosequencing. Int Microbiol. 2010;13(2):59–65. 10.2436/20.1501.01.111 20890840

[27] Medlin L, Elwood HJ, Stickel S, Sogin ML. The characterization of enzymatically amplified eukaryotic 16S-like rRNA-coding regions. Gene. 1988;71(2):491–9. 3224833 10.1016/0378-1119(88)90066-2

[28] Stiller JW, McClanahan A. Phyto‐specific 16S rDNA PCR primers for recovering algal and plant sequences from mixed samples. Molecular ecology notes. 2005;5(1):1–3.

[29] Team RC. R: A language and environment for statistical computing. 2013.

[30] Bae S, Wuertz S. Rapid decay of host-specific fecal Bacteroidales cells in seawater as measured by quantitative PCR with propidium monoazide. Water research. 2009;43(19):4850–9. 10.1016/j.watres.2009.06.053 19656546

[31] Nocker A, Mazza A, Masson L, Camper AK, Brousseau R. Selective detection of live bacteria combining propidium monoazide sample treatment with microarray technology. Journal of microbiological methods. 2009;76(3):253–61. 10.1016/j.mimet.2008.11.004 19103234

[32] Nocker A, Sossa KE, Camper AK. Molecular monitoring of disinfection efficacy using propidium monoazide in combination with quantitative PCR. Journal of microbiological methods. 2007;70(2):252–60. 10.1016/j.mimet.2007.04.014 17544161

[33] Pan Y, Breidt F. Enumeration of viable Listeria monocytogenes cells by real-time PCR with propidium monoazide and ethidium monoazide in the presence of dead cells. Applied and Environmental Microbiology. 2007;73(24):8028–31. 10.1128/AEM.01198-07 17933922 PMC2168130

[34] Emerson JB, Adams RI, Román CMB, Brooks B, Coil DA, Dahlhausen K, et al Schrödinger’s microbes: tools for distinguishing the living from the dead in microbial ecosystems. Microbiome. 2017;5(1):86 10.1186/s40168-017-0285-3 28810907 PMC5558654

[35] Lee DY, Rhee GY. Kinetics of cell death in the cyanobacterium Anabaena flos‐aquae and the production of dissolved organic carbon Journal of Phycology. 1997;33(6):991–8.

[36] Fittipaldi M, Nocker A, Codony F. Progress in understanding preferential detection of live cells using viability dyes in combination with DNA amplification. Journal of Microbiological Methods. 2012;91(2):276–89. 10.1016/j.mimet.2012.08.007 22940102

[37] Contreras PJ, Urrutia H, Sossa K, Nocker A. Effect of PCR amplicon length on suppressing signals from membrane-compromised cells by propidium monoazide treatment. Journal of microbiological methods. 2011;87(1):89–95. 10.1016/j.mimet.2011.07.016 21821068

[38] Luo J-F, Lin W-T, Guo Y. Method to detect only viable cells in microbial ecology. Applied microbiology and biotechnology. 2010;86(1):377–84. 10.1007/s00253-009-2373-1 20024544

[39] Nkuipou-Kenfack E, Engel H, Fakih S, Nocker A. Improving efficiency of viability-PCR for selective detection of live cells. Journal of microbiological methods. 2013;93(1):20–4. 10.1016/j.mimet.2013.01.018 23389080

[40] Soejima T, Schlitt-Dittrich F, Yoshida S-i. Polymerase chain reaction amplification length-dependent ethidium monoazide suppression power for heat-killed cells of Enterobacteriaceae. Analytical biochemistry. 2011;418(1):37–43. 10.1016/j.ab.2011.06.027 21771573

[41] Selvin R, Reguera B, Bravo I, Yentsch C. Use of fluorescein diacetate (FDA) as a single-cell probe of metabolic activity in dinoflagellate cultures. Biological oceanography. 1989;6(5–6):505–11.

[42] Nocker A, Camper AK. Novel approaches toward preferential detection of viable cells using nucleic acid amplification techniques. FEMS Microbiology Letters. 2009;291(2):137–42. 10.1111/j.1574-6968.2008.01429.x 19054073

[43] Desneux J, Chemaly M, Pourcher A-M. Experimental design for the optimization of propidium monoazide treatment to quantify viable and non-viable bacteria in piggery effluents. BMC microbiology. 2015;15(1):164. 26276157 10.1186/s12866-015-0505-6 PMC4537567

[44] Rappé MS, Kemp PF, Giovannoni SJ. Phylogenetic diversity of marine coastal picoplankton 16S rRNA genes cloned from the continental shelf off Cape Hatteras, North Carolina. Limnology and Oceanography. 1997;42(5):811–26.

[45] Rappé MS, Suzuki MT, Vergin KL, Giovannoni SJ. Phylogenetic diversity of ultraplankton plastid small-subunit rRNA genes recovered in environmental nucleic acid samples from the Pacific and Atlantic coasts of the United States. Applied and environmental microbiology. 1998;64(1):294–303. 9435081 10.1128/aem.64.1.294-303.1998 PMC124708

[46] Zwart G, Crump BC, Kamst-van Agterveld MP, Hagen F, Han S-K. Typical freshwater bacteria: an analysis of available 16S rRNA gene sequences from plankton of lakes and rivers. Aquatic microbial ecology. 2002;28(2):141–55.

[47] Betournay S, Marsh AC, Donello N, Stiller JW. Selective recovery of microalgae from diverse habitats using" phyto-specific" 16S rDNA primers. Journal of phycology. 2007;43(3):609–13.

